# Physicochemical Properties of a New Green Honey from Banggi Island, Sabah

**DOI:** 10.3390/molecules27134164

**Published:** 2022-06-29

**Authors:** Nanthini Rajindran, Roswanira Abdul Wahab, Nurul Huda, Norliza Julmohammad, Amir Husni Mohd Shariff, Norjihada Izzah Ismail, Fahrul Huyop

**Affiliations:** 1 Department of Biosciences, Faculty of Science, Universiti Teknologi Malaysia, Johor Bahru 81310, Johor, Malaysia; nanthinirajindran@graduate.utm.my; 2 Department of Chemistry, Faculty of Science, Universiti Teknologi Malaysia, Johor Bahru 81310, Johor, Malaysia; roswanira@kimia.fs.utm.my; 3 Faculty of Food Science and Nutrition, Universiti Malaysia Sabah, Kota Kinabalu 88400, Sabah, Malaysia; norliza@ums.edu.my; 4 Greentreebee Enterprise, Kuantan 26070, Pahang, Malaysia; amir.husni@gmail.com; 5 School of Biomedical Engineering and Health Sciences, Faculty of Engineering, Universiti Teknologi Malaysia, Johor Bahru 81310, Johor, Malaysia; norjihada@utm.my

**Keywords:** green honey, Sabah honey, Borneo, physicochemical, amino acid, sugar content

## Abstract

Green honey is exclusively available on the island of Banggi in Sabah, and its uniqueness sees the commodity being sold at a high market price. Therefore, green honey is prone to adulteration by unscrupulous individuals, possibly compromising the health of those consuming this food commodity for its curative properties. Moreover, an established standard for reducing sugar in green honey is unavailable. Ipso facto, the study aimed to profile green honey’s physical and chemical properties, such as its pH, moisture content, free acidity, ash content, electroconductivity, hydroxymethylfurfural (HMF), total phenolic content, total flavonoid content, DPPH, colour, total sugar content, total protein content, and heavy metals as well as volatile organic compounds, the data of which are profoundly valuable in safeguarding consumers’ safety while providing information for its quality certification for local consumption and export. The results revealed that the honey’s physicochemical profile is comparable to other reported kinds of honey. The honey’s naturally green colour is because of the chlorophyll from the nectar from various flowers on the island. The raw honey showed free acidity between 28 and 33 Meq/100 g, lower than the standard’s 50 Meq/100 g. The hydroxymethylfurfural content is the lowest compared to other reported honey samples, with the total phenolic content between 16 and 19 mg GAE/100 g. The honey’s reducing sugar content is lower (~37.9%) than processed ones (56.3%) because of water removal. The protein content ranged from 1 to 2 gm/kg, 4- to 6-fold and 2-fold higher than local and manuka honey, respectively. The exceptionally high content of trans-4-hydroxyproline in raw honey is its source of collagen and other healing agents. Interestingly, low levels of arsenic, lead, nickel, cadmium, copper, and cobalt were detected in the honey samples, presumably due to their subterranean hives. Nevertheless, the honey is fit for general consumption as the concentrations were below the maxima in the Codex Alimentarius Commission of 2001.

## 1. Introduction

**Figure 1:**
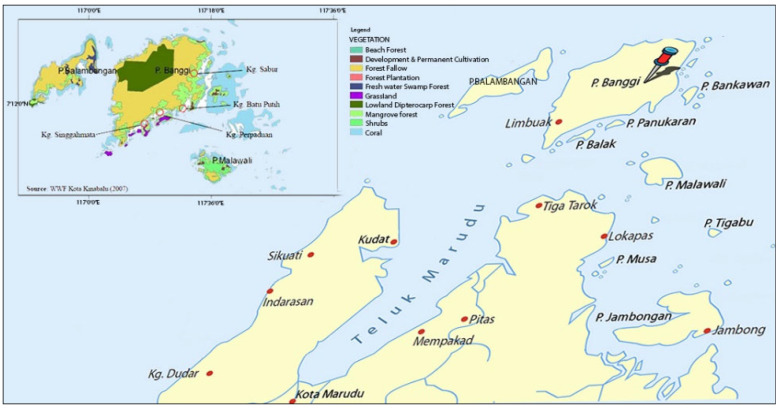
Location of Banggi Island at the tip of Borneo is in the South China Sea. The natural vegetation might be the source of the natural food ingredients for these honey bees on the island.

The existence of green honey is a boon to many people of Borneo, especially on Banggi Island (7.26° North, 117.15° East), which is located at the northern tip of Sabah as shown in Figure 1. The nearest mainland town to the island is Kudat, which is about 26 km away, and Kudat town itself is about 190 km from the north of Kota Kinabalu [1]. The green honey food substance made by the honeybee is produced in the underground space of the forest on Banggi Island. The honey from this island appears green and makes it different from other commonly seen brown honey [2]. It is assumed that the bee consumes algae or collects the nectar from the bamboo tree and this is the reason for honey’s green colour. Another assumption is that the green honey colour is because it might contain chlorophyll pigments. Green honey has a good marketing value since, as a natural product, its unique colour compared to other common honey gives consumers a different perspective, hence it increases the commercial value of the green honey [3]. The bee species have been tentatively identified as *Apis cerana* by an independent taxonomist. However, this needs to be clarified in separate studies and is not included in the present study.

There are a few important physicochemical properties such as pH, moisture content, HMF, and antioxidant properties, which are used to determine the quality of the honey [4]. The lower the pH, the lower the presence of microbial content because most of the microorganisms are unable to withstand acidic conditions. At the same time, the moisture content should be low as well because a highwater content promotes fermentation. HMF is used to determine the freshness of honey. Processing honey with a higher temperature or longer storage period can lead to a high HMF value [4]. Moreover, greater antioxidant properties are vital in biomedical activity. This study focuses on green honey from a different point of view. In this study, we determine the physicochemical properties of two types of green honey, namely, the commercially available bottled green honey (processed green honey) and freshly collected green honey from Banggi Island (raw green honey), where the broodless honeycomb was pressed to obtain the green honey. Honey is mainly composed of sugar components, especially fructose and glucose, followed by sucrose and maltose [5]. Sugar in honey is responsible for the viscosity, and the hygroscopic and granulation characteristics of the honey. However, the sugar content of the honey depends on the botanical and geographical regions. Honey is prone to adulteration by the direct addition of a certain amount of sucrose syrup into the honey and also the addition of chemical colour to attract consumers [6,7].

Generally, honey is rich in glucose and fructose and the percentage of sucrose in honey should be lower, which is less than 5% [8]. However, it is assumed that green honey contains higher sucrose content compared to glucose and fructose level. Interestingly, many decades ago, a certain honey, for instance, a sample collected from Australia, was reported to have a high sucrose content (11%) [9]. Then again, honey from different parts of the world has ingredients unique to the region, which contribute to varying medicinal properties as one of the benefits of honey consumption. That said, this study aims to identify the authenticity of the green honey as well identify the presence of any harmful substances, such as heavy metals, where it is sold in the local and international market. For this purpose, the nutritional and physicochemical properties of green honey were reported and compared to different types of commercially available honey in local Malaysian and international markets.

## 2. Results and Discussions

### 2.1. Physicochemical Properties

The physicochemical properties of Sabah green honey were compared to other common local honey, namely Tualang, Tualang Sabah, Gelam, and Kelulut, while Manuka was used as the standard for international honey. The pH, moisture content, free acidity, electroconductivity, HMF, total phenolic content, total flavonoid content, and antioxidant properties were analysed, and the overall results for physicochemical parameters are shown in Table 1. Raw green honey was collected fresh from the farm, whereas the processed green honey underwent dehydration (removal of water content) using a concentrator. The international standard and quality of commercial honey were determined for HMF, free acidity, and moisture content [8,10]. Electroconductivity and ash concentration were used as key indicators for determining the honey’s botanic origin [11]. This is because the amounts of mineral salts, organic acids, and protein in honey are related to the electroconductivity and ash levels. pH and electroconductivity were used to distinguish between honey samples from different geographical origins [12].

A. Moisture Content

Moisture content is the key parameter to identify the quality of honey because it can influence the crystallization, viscosity, palatability, specific weight, preservation, and flavour. A high volume of water in honey could result in fermentation, forming carbon dioxide and ethyl alcohol, which is not desirable [13,14]. The honey will taste sour with the increase in acidity from the acetic acid produced through the oxidation of alcohol that is formed by fermentation [15]. The consequences of this are that the quality of the honey is reduced significantly followed by the loss of flavour [16]. Honey with a high moisture content could potentially spoil quickly in comparison to a sample with a low moisture content, and the latter may have an extended shelf-life [17]. Thus, to prevent fermentation in the honey, the Codex Alimentarius (2001) and European Union Council (2002) stated that the moisture content in honey should not exceed 20%. From this study, the moisture content for both types of green honey ranged from 12.7 to 28.3% (Table 1). The raw green honey showed a moisture content of 22.5%, and is higher during the rainy season. For commercial and export quality, the raw green honey was further dehydrated down to 12.7% (Table 1), which meets the criteria of the Codex Alimentarius (2001). The water content of the processed green honey (12.7%) is lower than the water content in Manuka honey (19.5%). The highwater content in raw green honey corresponded to the moisture content normally observed for honey produced in tropical countries, such as Malaysia, between 18.70 and 35.7% [18]. Some factors have been identified as reasons for the high moisture content in tropical honey including the maturity level in the hive, harvesting season, and the weather conditions, alongside the all year round heavy rainfall in Malaysia. The heavy rainfall in particular contributes to the high humidity that affects the moisture content of the raw green honey. Moreover, the bees produced the green honey in underground soil, which retains a lot of water, particularly during the rainy season. Most of the local honey products in Malaysia are predicted to surpass the maximum moisture content, and must be dehydrated to reduce the moisture for an improved shelf life.

B. pH

All honeys studied were found to be acidic with their pH values ranging between pH 3 and 4 (Table 1). The pH values for raw green honey and processed green honey were 3.4 ± 0.03 and 3.7 ± 0.02, respectively; lower than the pH of Manuka honey (pH 4.03 ± 0.2), while Kelulut honey was the most acidic. According to Bogdanov et al. [19], honeydew honey has a pH between 4.5 and 6.5, while the pH of blossom honey is between 3.5 and 4.5. From this study, it was found that only processed green honey and Manuka honey showed pH values close to pH 4, while other honey samples were of lower values than those reported for blossom honey. Storage conditions and harvesting states could also be the reasons behind the varying pH values [20] in addition to the differences in bee species [21]. On the other hand, none of the tested honey samples showed pH values similar to the honeydew honey (pH 3.5 to 4.5). It can be concluded that all the honeys used in this study were blossom honey, made of plant nectar collected by bees.

Another importance of the acidity of honey is its antibacterial property. The acidity of honey is vital for preventing the growth of most pathogenic microorganisms [22]. Natural organic acid in honey is one of the contributors to the low pH of honey [23,24]. Nevertheless, preventing bacterial spoilage also extends the shelf life. All the analysed honey showed good antibacterial properties based on their pH values, especially the raw green honey, Tualang, Kelulut, and Gelam, which showed lower pH values. The low pH in honey is compatible with naturally acidic foods, which also contributes to the product’s flavour profile. In short, pH plays an important role, especially during honey extraction and for long-term storage, because it affects the shelf life of the honey.

C. Free acidity

The free acidity for all honeys in this study ranged from 28.0 to 448.1 meq/kg (Table 1). Kelulut honey showed the highest free acidity value of 448.10 meq/kg [4], while processed green honey had the lowest free acidity corresponding to 28.0 meq/kg. According to the Codex Alimentarius [8] and European regulations [10], free acidity of honey must be less than 50 meq/kg. After the honey extraction from combs, the time of extracting and conditions under which honey is stored could affect the free acidity value in honey. Free acids induce a sour taste in honey. A low free acidity value demonstrates a low level of undesirable fermentation due to a high moisture content. The rate of conversion from fructose and glucose to ethyl alcohol and carbon dioxide by osmotolerant yeast and the further formation of acetic acid and water in the presence of oxygen is relatively low, resulting in a small free acidity value [25]. Honey samples with a high concentration of free acidity also imply the presence of internal esters, lactone, and inorganic ions such as chloride, sulphate, and phosphate in equilibrium with organic acids [26].

D. Ash content

Ash contents for the tested Malaysian honey samples were found to be between 0.08 g/100 g and 4.4 g/100 g (Table 1). Manuka honey, which acts as the international standard honey, had an ash content of 0.30 g/100 g, lower than the processed green honey. The ash content is a direct measure of the inorganic residues of honey after carbonization [27]. Another important use of this parameter is for predicting trace elements and minerals essential to a healthy diet (e.g., calcium, phosphorous, potassium, and sodium). Honey samples with a high ash content convey an abundance of trace elements and minerals [28]. Therefore, among all the honey samples, it was expected that the processed green honey, which has the highest ash content, would possess the highest amount of minerals. According to the Codex Alimentarius standard, ash content for honeydew honey should be below 1.2 g/100 g, while it is lower than 0.6 g/100 g for blossom honey [29]. However, the ash content for the processed green honey (4.4 ± 0.01) was well above these values. This is because the naturally abundant minerals and trace elements in raw green honey become concentrated upon dehydration to yield the processed honey.

E. Electroconductivity

Generally, electroconductivity is one of the main specifications used regularly to check the quality of honey. It is an excellent parameter for identifying the pureness of honey and its botanical origin [30]. Among others, honey contains components such as organic acids and minerals, which, when in an aqueous solution, dissociate into ions with electroconductive capability [30]. Concentrations of protein, organic acids, and mineral salts directly correlate with the electroconductivity of honey and can be used for honey classification of various floral origins, too [12,26]. It is common for honey to have a low concentration of organic acids, despite organic acids being considered as minor elements. The content of such acids is one of the main contributing factors to the honey’s chemical, physical, and organoleptic properties [31]. Moreover, organic acid content greatly varies according to the botanical origin [32]. The acid and ash content can appreciably influence the electroconductivity in honey. Higher conductivity shows that the honey has a larger amount of acid and ash and it can be seen from the honey’s colour. The literature has shown that darker coloured honey samples have higher electroconductivity. Other factors, such as the storage time and floral source can also affect the electroconductivity of the honey [33]. Codex and EU standards set EC values of ≤0.8 mS/cm for honeydew honey and ≥0.8 mS/cm for blossom honey [8,10]. From the analysis, raw and processed green honey has the highest electroconductivity corresponding to 1.93 mS/cm and 0.85 mS/cm, respectively. This indicates that green honey has higher amounts of organic acids, mineral salts, and proteins than the other types of honey investigated in this study. Tualang (0.83 mS/cm) and Kelulut (1.05 mS/cm) honey samples also have higher electroconductivity values than the international limit (≤0.8 mS/cm for honeydew honey; ≥0.8 mS/cm for blossom honey), whereas, Gelam honey (0.43 mS/cm) and Manuka honey (0.53 mS/cm) showed lower electroconductivity than the international limit. In short, it can be seen that most of the honey collected in Malaysia has a higher electroconductivity, which correlates with their high mineral content [34]. Studies have shown that the electroconductivity of honey is inversely correlated with the content of plant pollen [35].

F. Hydroxymethylfurfural (HMF)

The presence of HMF in honey determines the freshness of the honey in general. HMF is an organic molecule generated when fructose is broken down in an acidic environment via the Maillard reaction. In this study the HMF contents in the tested honey samples ranged from 0.01 to 63.42 mg/kg honey (Table 1). Pertinently, the HMF content of tropical honey must not exceed 80 mg/kg honey. Honey that has been stored and pasteurised for a long time might have a higher HMF content, while fresh, unheated, and non-adulterated honey has low HMF values [8]. In this work, the HMF values for the raw and processed green honey were lower than other honeys, with values of 0.01 mg/kg and 0.6 mg/kg, respectively. The results seen here were acceptable for Kelulut honey (HMF value 45.93 mg/kg), but Gelam, Tualang, and Manuka honeys showed appreciably higher HMF content (45.42 ± 0.02 to 63.42 ± 0.01 mg/kg) (Table 1). HMF values of less than 1 mg/kg in fresh honeys tend to increase with increasing temperature and duration of the heating, and extended storage conditions [6,36]. Commercial honey is frequently processed in a variety of ways, such as filtration and evaporation, to increase the shelf life and maintain the product’s quality [37]. That said, the HMF value is a parameter used to calculate the acceptable duration of honey before it becomes unfit for consumption. Regardless of the honey type, it is strongly recommended that the honey is consumed within six months to one year after harvesting or processing.

G. Total Phenolic Content (TPC)

Honey’s antioxidant activity is mostly attributed to phenolic chemicals, and such chemicals protect cells from damage by free radicals [38]. The TPC in honey varies according to plant types, which are influenced by the climate, humidity, and excessive solar exposure. Sun-exposed plants usually have significantly higher TPC than shade-grown species [39,40,41]. In this assessment, the TPC of processed green honey and raw green honey was 15.57 mg GAE/100 g and 18.95 GAE/100 g, respectively; slightly higher value than in Gelam. However, higher TPCs were observed for raw Manuka (1288.0 mg GAE/100 g), Tualang (31.84 ± 0.76 mg GAE/100 mg), and Kelulut (42.6 ± 1.4 GAE/100 mg) honeys. Although the TPC values in both raw and processed green honey were lower, this difference is somewhat expected considering the different geographical nature of the honey source, as well as the foraging preference of the soil-residing bees that produce green honey.

H. Total Flavonoid Content (TFC)

Pollen, nectar, or propolis are the main sources of flavonoids. The low molecular weight of flavonoids influences the aroma and antioxidant qualities of honey [42]. The most common flavonoids found in honey include pinocembrin, apigenin, kaempferol, quercetin, pinobanksin, luteolin, galangin, hesperetin, and isorhamnetin, with the flavanol quercetin being the major compound. These compounds have been reported to exhibit good curative properties against certain diseases [43,44], while flavanols are used as standard markers for identifying the floral and geographical origins of honey [45,46]. From the analysis, it was observed that the TFC values for the Malaysian honey samples were between 4.58 ± 0.01 and 58.3 ± 1.60 mg QE/100 g. The raw and processed green honey had lower TFC values of 7.6 mg QE/100 g and 4.58 mg QE/100 g, respectively, compared to Manuka honey, which had a TFC within a moderate range (37.64 mg QE/100 g).

I. TDPPH

The DPPH (2,2-diphenyl-1-picrylhydrazyl) method, FRAP (ferric-reducing/antioxidant power) assay, ORAC (oxygen radical absorbance capacity) assay, and TEAC (Trolox equivalent antioxidant activity) assay are typical methods used to estimate the antioxidant activity of honey. Meanwhile, the DPPH test solely measures the activity of water-soluble antioxidants [47]. The antioxidant potential of honey has been found to be closely associated with its phenolic acid and flavonoid content [47,48,49]. The DPPH method is popular for this reason because of its stable radical component, and it is a straightforward assay to measure the scavenging activity of a compound [50]. Table 1 shows the DPPH values of the raw and processed green honey corresponding to 2.3% and 2.0%, respectively, but the Gelam honey was found to be the highest at 76.29%. The outcome seen here correlated well with the low concentrations of phenolic compounds and flavonoids in both raw and processed green honey, and therefore the lower percentage of radical-scavenging activity.

J. Chlorophyll

**Figure 2:**
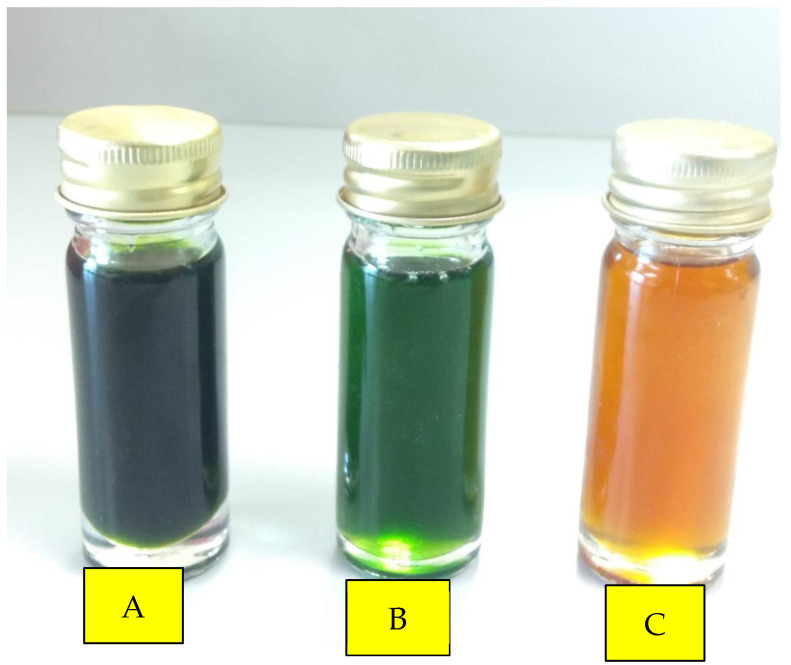
The green honey and common brown honey originating from Banggi Island, Sabah. Processed green honey (**A**) is darker than raw green honey (**B**). A common brown colour of raw Tualang honey (**C**).

The honey’s colour is the first aspect of its quality that influences consumer preference. The different colours in honey, ranging from colourless to dark/brown, imply the presence of antioxidant pigments apart from the nectar composition, as well as differences in the process acquisition, temperature, and storage duration [48]. Pertinently, the unique green coloured honey from Banggi Island of Sabah is a stark difference from the amber-coloured Manuka and Malaysian honey samples. The difference in the former is due to the honey’s unique high chlorophyll contents of 41 mg/kg and 539 mg/kg in the processed and raw green honey, respectively (Figure 2). The result seen here implies the Banggi Island’s bees peculiar foraging behaviour of collecting chlorophylls from the surrounding bamboo forest. Conversely, coloured honey was reported in France in 2012, but further investigation revealed that the green-coloured honey was due to the honey bees foraging on candy coating from a nearby plant that processes M&M factory waste (https://inhabitat.com/waste-from-mms-candy-causes-honey-to-turn-green-in-france, accessed in 26 July 2012), whereas the green-coloured honey found on Banggi Island was believed to be contributed to by natural sources, i.e., specialised plant species found on the island. However, further research is required to validate this hypothesis.

### 2.2. Sugar Content

Fructose and glucose are common sugars in honey, followed by maltose and sucrose [51,52]. However, the sugar components in the green honey showed sucrose and glucose, followed by fructose, as being the major sugars. In comparison, the total sugar content in all Malaysian honey samples ranged between 39.30 g/100 g and 64.19 g/100 g, with Manuka having the highest total sugar content (64.19 ± 2.69 mg/100 g) followed by Gelam (62.74 ± 0.06 mg/100 g), Kelulut (62.70 ± 0.12 mg/100 g), and raw Tualang (38.60 ± 0.02 mg/100 g) (Table 2). The dominant sugar profile was also different between the honey samples: where the Kelulut honey is high in glucose, Manuka honey has high fructose and maltose contents, and the green honey is high in sucrose. These discrepancies can be explained by the different botanical and geographical regions, weather, and the post-harvest factors such as storage and processing conditions [51,52]. Most importantly, the study outcome indicated that all the Malaysian raw honey samples adhered to the international standard for sugar content, with the sums of fructose and glucose contents not less than 60%. The high levels of sucrose in honey could be attributable to the invertase enzyme, which does not fully hydrolyse the sucrose in nectar or honeydew, or possibly due to underdeveloped honey, a high nectar flux, or bee artificial feeding [53,54].

Honey with a higher proportion of fructose than glucose can be distinguished from the commercial invert sugar technique [56]. The fructose content in honey should exceed the glucose amount in order for it to be of good quality [57]. This is due to the fact that glucose is less water soluble than fructose. If the glucose level is higher than the fructose amount, it is more likely to crystallise, thus lowering the honey quality [29]. The glucose/water (G/W) ratio of Malaysian raw honey was lower than in Manuka honey. A low G/W ratio slows the pace of sugar crystallisation. Aside from that, due to the lower fructose/glucose (F/G) ratio, Malaysian raw honey is less sweet than Manuka honey [5,58]. At present, the sucrose content of the processed green honey is higher compared to glucose and fructose. For commercial purposes, the sucrose level of honey should not exceed 5 g/100 g. Raw and processed green honey had a sucrose content of 14.55 g/100 g and 27.72 g/100 g, respectively. The unusually high sucrose content seen here suggests the collection of underdeveloped honey due to aggressive harvesting of the wild bees’ honey or the bees’ preference on certain plant nectar with high sucrose contents.

Similarly, the sugar level of certain Australian honey has been observed to be high as well, where honeys obtained directly from the combs in hives of bees that had never been fed showed abnormally high quantities of sucrose. This proved that the high sucrose content was due to the natural honey produced by these bees rather than adulteration or because the harvesting was performed during a hot and dry season [9]. In Australia, plant species from the heath-like understory or plants that grow on deep infertile sands of the coastal plain contributed to the high sugar levels in nectar [9]. Additionally, the higher sucrose content in honey is also influenced by the type of pollinator or bee. Sucrose dominates nectar sugar composition in species pollinated by hummingbirds, moths, and long-tongued bees, whereas hexoses dominate nectar sugar composition in species pollinated by passerines, short-tongued bees, and neotropical bats [59]. Therefore, plant species from regions with a high temperature might produce sucrose-dominant nectar. To confirm this, a future metagenomic study on green honey might be necessary.

### 2.3. Proteins and Amino Acids

Protein and amino acid contents in honeys can differ from one botanical or geographical origin to another, and are affected by storage duration. The main protein constituents in honey are enzymes [14], which partake in the ripening of honey, thus producing higher protein levels. The level of total protein in honey typically ranges from 2000 to 5000 mg/kg [60]. The protein content in Malaysian honeys ranged from 399.81 mg/kg to 6000 mg/kg (Table 3). Raw Tualang honey (Tualang Sabah) contained the highest amount of protein (6000 mg/kg), followed by green honey (2000 ± 17 to 4000 ± 18 mg/kg) and Manuka (1100 ± 18 mg/kg). The varying total protein contents could be due to differences in the floral source as well as the geographical origins of the honey.

Proline is an essential amino acid that is primarily synthesised by bees’ salivary secretions during the nectar-to-honey conversion process [61], and the content is used as an indicator of honey ripeness. Proline is the most common amino acid found in pollen grains and honey. The literature has shown that honeys with a high proline content have a lower probability of being adulterated [60]. In Manuka honey, the L-proline content was only 616.37 mg/kg. Our findings showed that proline is absent in green honey, presumably related to worker bees collecting pollen and nectar at the start of the flowering season, implying the collection of underdeveloped or immature honey [62]. Proline values in previously reported honeys such as Tualang, Gelam, and Manuka exceeded the globally acknowledged requirement of 180 mg/kg for proline [60].

On the other hand, green honey is rich in *trans*-4-hydroxyl-proline (2960.0 ± mg/kg), L-tryptophan (842.1 mg/kg), L-leucine (125.8 mg/kg), L-histidine (57.1 mg/kg), L-glycine (82.8 mg/kg), L-glutamic acid (36.1 mg/kg), and L-aspartic acid (37.8 mg/kg). To our knowledge, this is the first study to report the high content of trans-4-hydroxyl-proline in green honey. This non-essential amino acid has an important role in collagen synthesis and stabilization of the collagen triple helix [63]. Meanwhile, L-tryptophan is an amino acid needed for growing bacteria and a component of various plant propagation germination media. Tryptophan is a one-of-a-kind amino acid that is required for human survival, despite its low concentration in the human body. In fact, this amino acid is a vital component of many metabolic processes [64]. L-leucine is also an essential amino acid that aids in the treatment of obesity and diabetes, and a key amino acid in reducing tumour aggressiveness and metastatic sites [65]. On the one hand, L-histidine partakes in immunological responses, for instance, for the treatment of rheumatoid arthritis, allergic illness, ulcers, and anaemia induced by kidney failure or dialysis. L-histidine stimulates histamine production by stomach enterochromaffin cells and most other cells [66]. Conversely, L-glycine is required for growth, tissue maintenance, and the production of hormones and enzymes. The amino acid improves thinking skills and reduces schizophrenia after a stroke. The body converts L-glutamic acid to glutamate, which facilitates signal transmissions between nerve cells in the brain and to other cells. This amino acid plays a critical role in children’s brain development, and is one of the most important brain neurotransmitter messengers [67]. L-aspartic acid stimulates the creation of antibodies that help the immune system function [68]. Therefore, green honey has three essential amino acids for maintaining health and wellness.

### 2.4. Heavy Metals

The green honey from Banggi Island is harvested from underground nests built by the wild bees, hence there is the possibility of a higher presence of metal contaminants in this food commodity. Cadmium and lead are the two heavy metals used as bioindicators for honey contamination [60]. The results revealed that the concentrations of arsenic, lead, nickel, cadmium, copper, and cobalt in green honey were less than 0.5 mg/kg, attributed to the honey’s clean habitat (Table 4). Heavy metals were not detected, suggesting that Banggi Island is pollution free and the green honey is safe for consumption. This analysis is important in confirming the safety of this food commodity as there are reports on honeys from certain regions of the world containing heavy metal contaminants due to industrial pollution [69].

**Table 3:** Total protein and amino acid profiling of green honey.

Type of Honeys	Raw Green	Processed Green	Raw Tualang	Tualang [4]	Gelam [70]	Kelulut [4]	Manuka
Total protein content (mg/kg)	4000 ± 18	2000 ± 17	6000 ± 10	399.81 ± 0.10	644.08 ± 0.17	479.90 ± 0.24	1100 ± 0.31
L–alanine (mg/kg)	ND	ND	ND	NA	35.96 ± 0.26	NA	28.32 ± 0.20
L–arginine (mg/kg)	ND	ND	685.2 ± 0.26	NA	ND	NA	8.86 ± 0.23
L–asparigine (mg/kg)	ND	ND	ND	NA	ND	NA	19.15 ± 0.22
L–aspartic acid (mg/kg)	37.8 ± 0.25	ND	ND	NA	ND	NA	19.15 ± 0.20
L–cysteine (mg/kg)	ND	ND	392.8	NA	33.01 ± 0.22	NA	ND
L–glutamic acid (mg/kg)	36.1 ± 0.26	10.1	ND	NA	29.77 ± 0.23	NA	13.34 ± 0.22
L–glutamine (mg/kg)	ND	ND	ND	NA	29.32 ± 0.26	NA	13.34 ± 0.21
L–glycine (mg/kg)	82.8 ± 0.20	106.6 ± 0.21	ND	NA	ND	NA	11.58 ± 0.11
L–histidine (mg/kg)	57.1 ± 0.21	ND	ND	NA	17.82 ± 0.22	NA	16.13 ± 0.19
L–isoleucine (mg/kg)	ND	ND	ND	NA	6.38 ± 0.26	NA	8.36 ± 0.11
L–leucine (mg/kg)	125.8 ± 0.29	30.0 ± 0.20	403.7 ± 0.27	NA	44.50 ± 0.29	NA	7.68 ± 0.12
L–lysine (mg/kg)	ND	188.0 ± 0.23	172.9 ± 0.22	NA	14.67 ± 0.27	NA	40.86 ± 0.24
L–methionine (mg/kg)	ND	ND	137.9 ± 0.33	NA	19.49 ± 0.22	NA	0.67 ± 0.16
L–phenylalanine (mg/kg)	ND	ND	107.2 ± 0.36	NA	237.37 ± 0.22	NA	77.98 ± 0.36
L–proline (mg/kg)	ND	ND	1386.9 ± 0.44	NA	48.91 ± 0.19	NA	616.37 ± 0.21
L–serine (mg/kg)	ND	ND	ND	NA	19.07 ± 0.11	NA	21.60 ± 0.26
L–threonine (mg/kg)	ND	ND	ND	NA	7.07 ± 0.13	NA	9.23 ± 0.28
L–tryptophan (mg/kg)	842.1 ± 0.33	723.6 ± 0.41	131.0 ± 0.32	NA	19.05 ± 0.12	NA	3.33 ± 0.11
L–tyrosine (mg/kg)	ND	ND	ND	NA	19.57 ± 0.18	NA	16.38 ± 0.21
L–valine (mg/kg)	ND	ND	ND	NA	5.07 ± 0.16	NA	11.36 ± 0.26
Trans-4-hydroxyl-proline (mg/kg)	2960 ± 0.56	1102.6 ± 0.36	2972.2 ± 0.46	NA	29.63 ± 0.23	NA	9.53 ± 0.11

Note: All values are expressed as mean ± standard deviation of three replications. ND: not detected; NA: not available/not tested.

### 2.5. Volatile Organic Compound (VOC)

Honey’s organoleptic and nutritional qualities are attributed to volatile organic compounds, which make up a smaller portion of the overall composition. Aldehydes, ketones, acids, alcohols, hydrocarbons, norisoprenoids, terpenes, and benzene compounds, and their furan and pyran derivatives, are among the volatile substances found in honey [46,71]. In this study, few volatile organic compounds were detected in processed green honey, which were ethyl acetate, propanamide, N,N-dimethyl-, D-limonene, 1,4-cyclohexadiene, 1-methyl-4-(1-methylethyl)-, cyclohexene, 1-methyl-4-(1-methylethylidene)-, and benzene, (2-methyl-1-propenyl)-*O*-Isopropenyltoluene (Table 5). Conversely, ethyl acetate was the only volatile organic compound that is detected in Sabah Tualang raw honey.

Ethyl acetate is the most common ester in fruits, and is a fruity-smelling liquid with a brandy undertone [72] that substantially impacts distillate organoleptic properties. Ethyl acetate is also a common ingredient in food and beverages. It is found in artificial fruit essences, sweets, baked goods, gums, ice creams, and confectionary as an artificial flavouring agent.

D-limonene is a terpene found in abundance in nature, which is clinically used to dissolve cholesterol-containing gallstones. The compound can relieve heartburn and gastroesophageal reflux due to its gastric acid neutralising effect and supports proper peristalsis (GERD). D-limonene also has chemopreventive properties against a variety of cancers [73] and, in some cases, could lessen inflammation [74,75]. Apart from anticancer properties, limonene lowers blood cholesterol, sugar, and triglyceride levels [76].

Honey’s volatile chemicals have been observed to be considerably affected by overheating during processing or long-term storage [46,53], the latter of which changes honey’s composition, i.e., the percentage of volatile compounds produced by certain aromatic plant species, namely, *Foeniculum vulgare* Mill. [77]. *Foeniculum vulgare* contains propanamide, D-limonene, benzene, (2-methyl-1-propenyl)-o-Isopropenyltoluene and cyclohexene, and 1-methyl-4-(1 methylethylidene) [78]. The aroma in combination with taste and physical components contribute to the unique honey flavour [79]. This is because volatile compounds in honeys are a complex mixture of minute compounds with varying physicochemical qualities and stability. Therefore, their detection requires suitable techniques for their identification and quantification in the green honey [80].

## 3. Materials and Methods

### 3.1. Honey Sample

Processed and raw green honey were obtained from NS Field Sdn. Bhd., Sabah for the analysis. Two types of honey were analysed, (a) the commercially available green honey (processed green honey) and (b) freshly collected green honey (raw green honey). The broodless honeycomb was pressed to obtain the honey. All honey samples were kept in a screw-capped dark container stored at room temperature (approximately 25 °C) prior to analyses.

### 3.2. Materials

Milli-Q-ultrapure water, sodium hydroxide (NaOH), sodium carbonate (Na_2_CO_3_), copper sulphate, (CuSO_4_), sodium citrate, Bovine Serum Albumin (BSA), potassium ferrocyanide (K_4_Fe (CN_6_)·3H_2_O), zinc acetate (ZnCH_3_COO_2_·2H_2_O), sodium bisulphite (NaHSO_3_), ethanol, sodium nitrite, sodium acetate (C_2_H_3_ NaO_2_), iron (II) sulphate (FeSO_4_·7H_2_O), and acetic glacial (CH_3_COOH) were purchased from R&M Chemicals (Essex, UK). 2,4,6-Tri(2-pyridyl)-s-tiazine (TPTZ) and 2,2-diphenyl-1-hydrazyl-hydrate (DPPH), sodium carbonate (Na_2_CO_3_), aluminium chloride (AlCl_3_) and all standards for organic acids (gluconic, formic, tartaric, D-malic, citric, acetic, succinic, and lactic acid), 5-hydroxymethylfurfural (HMF), perfluorooctanoic acid (96%), sugars (fructose, glucose, sucrose, maltose), Gallic acid, quercetin, ascorbic acid were purchased from Sigma-Aldrich (St. Louis, Missouri, United State). Iron (III) chloride hexahydrate (FeCl_3_·6H_2_O), Folin–Ciocalteu’s phenol reagent, sodium sulphate anhydrous (Na_2_SO_4_), ethyl acetate (CH_3_COOC_2_H_5_, 99.8%), formic acid (CH_2_O_2_, 98%), metaphosphoric acid (EMSURE^®^), acetonitrile (ACN), methanol (CH_3_OH), hydrochloric acid fuming (HCl, 37%) were purchased from Merck (Darmstadt, Germany). Sulphuric acid (H_2_SO_4_-96.5%) was purchased from SIGMA. The water was purified using ELGA Pure Lab Classic system (ELGA, Woodridge, IL, USA). All chemicals and solvents used were of analytical grade except for HPLC analysis.

### 3.3. Physicochemical Properties

The pH of green honey samples was assessed according to the AOAC method 962.19 (AOAC, 2016). A waterproof Mettler Toledo pH meter (Switzerland) was used to measure the pH value.

A. Moisture Content

The moisture contents of honey samples were analysed based on refractometric method established by International Honey Commission (IHC). The moisture was measured using a digital refractometer (Digital ATAGO™ RX-5000α, Germany) at 20 °C. The RI value was converted to the moisture content (%) using Equation (1) [81]

Moisture content (%) = [−0.2681 − log (R1 − 1)/0.002243] (1)

B. Free acidity

Acidity is determined in accordance with the method described by International

Honey Commission Method (IHC) [82]. The titration was carried out with NaOH 0.1 N, until the solution reached a pH of 8.5. NaOH (10 mL) was added to the sample to increase the pH to approximately 10. It was titrated with HCl (0.1 N) to slowly return the pH to 8.3. The volumes spent during each titration were noted to calculate the total acidity of the sample. Acidity value was determined by Equation (2).

Free acidity: corrected volume of NaOH spent × 10(2)

C. Ash content

The ash was determined using a Phoenix electrical furnace (CEM, Charlotte, NC, USA) adjustable up to 600 °C for one hour (±25 °C). Weight loss occurred when the product was incinerated, resulting in the destruction of the organic matter without changing the constituents of the mineral residue or causing loss by volatilization [8]. The resulted ash was weighed and expressed as ash content in g/100 g honey.

D. Electroconductivity (EC)

Honey solution of 20% (*w*/*v*) was prepared by dissolving 20 g of honey in 100 mL ultrapure water prior to measuring the electroconductivity using HI-98311 electroconductivity meter (Hanna Instruments, Woonsocket, RI, USA). Green honey samples were analysed in triplicate.

E. Sugar Content

The quantification of sugars was performed using High Performance Liquid Chromatography equipped with Refractive Index Detector (HPLC-RID) following the protocol by AOAC Official Method 977.20 [83]. A small amount of 5% of honey (*w*/*v*) was dissolved in a mixture of distilled water and acetonitrile (1:1), filter sterilized through 0.22 µm nylon syringe filter. Zorbax Carbohydrate Column (Agilent, Santa Clara, CA, USA) with 5 μm particle size, 250 mm in length, and 4.6 mm inner diameter was used as stationary phase. An isocratic elution mobile phase consisting of acetonitrile and distilled water was used in a ratio of 75:25. An aliquot of 40 μL of honey sample was injected with a flow rate of 1.4 mL/min with the column temperature maintained at 30 °C. Run time per sample was set at 25 min matched to standard glucose, fructose, maltose, and sucrose. The sugar content was recognized by comparing them with the retention time of standard sugars. Quantitative analyses were performed by preparing standard solutions of fructose, glucose, sucrose, and maltose at different concentrations. Subsequently, the calibration curves were constructed using the respective peak area of analytes.

F. Protein Content

The reagents used were prepared as follows: (1) Reagent A: 20 g of sodium carbonate, Na_2_CO_3_ was mixed with 4 g of sodium hydroxide and made up to 1 L with distilled water. The reagent was stored in 4 °C. (2) Reagent B: 0.5 g copper sulphate, CuSO_4_ was mixed with 1 g sodium citrate and made up to 100 mL distilled water. The reagent was stored in 4 °C. (3) Reagent C was freshly prepared by mixing 50 mL of Reagent A and 1 mL of Reagent B. (4) Reagent D: Folin–Ciocalteu reagent was freshly prepared by diluting with distilled water in the ratio of 1:1. Protein content in the honey samples was measured using a Lowry method described by International Honey Commission (IHC) [82]. An aliquot of 0.2 mL (0.1 g/mL) of each honey sample was mixed with 2 mL of Reagent C and mixed well. The solutions were incubated at room temperature for 10 min followed by the addition of 0.2 mL Reagent D. Next, the mixtures were incubated at room temperature for another 30 min prior to measurement of A_660nm_. Bovine serum albumin (BSA) solutions were prepared in the range of 0.05−1.00 mg/mL and used as the standard for plotting a calibration curve. The protein concentration was expressed in mg of protein per kg of honey (mg/kg).

G. Hydroxymethylfurfural (HMF) Content

HMF content in green honey was determined as per method outlined by White and Doner [29] using spectrophotometric assays. Honey (5 g) was diluted with 25 mL distilled water and transferred into a 50 mL volumetric flask. A 0.5 mL Carrez solution I was added followed by 0.5 mL Carrez solution II. Distilled water was then added up to 50 mL. The solution was then filtered using a 0.45 µm filter paper, discarding the first 10 mL filtrate. Then, 5 mL honey solution was mixed with 5 mL water before measuring its absorbance against reference solution of 5 mL initial honey solution and 5 mL 0.2% sodium bisulphite solution. The absorbance was measured at 284 nm and 336 nm, and the values were expressed in (mg/kg).

H. Total Phenolic Content (TPC)

The Folin–Ciocalteu assay was designed and standardized for the quantification of total phenols by Singleton et al. [84] and Daves [85]. Total phenol content was determined by interpolating the absorbance of the honey based on a calibration curve constructed with standard Gallic acid, with a purity of 98%.

I. Total Flavonoid Content (TFC)

Total flavonoid concentration was determined by the method of Alothman et al. [86], using quercetin as standard (Sigma-Aldrich^™^) at 95% purity. The values were calculated as the total flavonoid concentration in quercetin equivalent/100 g of honey ± standard deviation.

J. DPPH Radical Scavenging Activity

The scavenging activity of honey samples for 2-2-diphenyl-1-picrylhydrazyl (DPPH) was measured as described by Ferreira et al. [87]. Initially, DPPH reagent was prepared with a concentration of 0.02 mg/mL in methanol. Honey samples were dissolved in ultrapure water with a concentration ranging from 5 to 60 mg/mL. For the test sample, 0.75 mL of each honey concentration was mixed with 1.5 mL of DPPH reagent. The radical scavenging activity (RSA) was calculated as the percentage of DPPH discoloration using the following equation

(3) $$\mathrm{RSA}\;(\%)=[(A_{\mathrm{DPPH}}-A_{S})/A_{\mathrm{DPPH}}]\times 100$$

K. Amino acid profiling

The honey (1 g) was transferred into a 50 mL tube with a cap. A volume of 9.95 mL of 0.1% (*v*/*v*) acetic acid and 50 μL of the internal standard working solution was added to the samples and the mixture was vortexed for 2 min. The solution was filtered through 0.45 μm pore size nylon syringe filter prior to LC–MS analysis. The Agilent 1200 series (Agilent, Technologies, Waldbronn, Germany) chromatograph coupled with Agilent 6410 Triple Quad LC/MS detector (Agilent Technologies, Palo Alto, CA, USA) was used for separation of free amino acids. Mobile phase was a mixture of 100 mL acetonitrile (Sigma-Aldrich, Steinheim, Germany), 1 mL of acetic acid (Fischer Scientific, Loughborough, UK), and 500 mL of 0.05 mM water solution of perfluorooctanoic acid (96%) (Sigma-Aldrich, Steinheim, Germany). Separation was achieved with Purospher Star RP-8ec column (150 mm × 94.6 mm × 93 μm; Merck, Darmstadt, Germany) at temperature of 25 °C and flow rate of 0.5 mL/min.

Detection was conducted in MRM mode based on the protonated molecular ions of the amino acids and the internal standard (standard mix solution), as well as collision-induced production of amino acid-specific fragment ions. The following instrumental parameters were used for LC–MS/MS analysis of amino acids in the positive MRM mode: nitrogen as a drying gas (320 °C) with the flow rate of 8.0 L/min, nebulizer pressure 50 psi, and capillary voltage 3 kV with the specific MRM amino acid transitions as well as their corresponding fragmentor voltages, collision energies and dwell [88]. Mixed stock solution of amino acids and a stock solution of an internal standard were prepared in concentration 1000 µg/mL of acidified water (0.1% acetic acid). Working solutions of amino acids and the internal standard prepared by 10× dilution of stock solutions in acidified water was 100 µg/mL. A calibration curve of amino acids was prepared in the range from 0.02 to 6 µg/mL, internal standard concentration was 0.5 µg/mL prepared in acidified water [89].

L. Chlorophyll

Each sample (triplicate) of green honey was prepared as described by Lichtenthaler and Buschmann [89]. The highest absorbance spectrum of chlorophyll was measured with a specific wavelength range between A_300–700nm_. The result was obtained in the form of chlorophyll absorption spectrum. The chlorophyll content in green honey was calculated using equation

Total chlorophyll (%) = [(7.12 × A_660nm_) + (16.8 × A_642nm_)] × 0.1 × 100/weight of sample (4)

M. Heavy metals

HNO_3_, H_2_O_2_, NaOH, and KHP were of analytical grade (BDH, England). Stock solutions (1000 mg/L concentration) of Cd, Pb, Cu, Mn, Ni, Cr, and Zn were from Inorganic Ventures (Christiansburg, VA, USA). The honey was analysed for the level of the selected heavy metals (Cd, Pb, Cu, Mn, Ni, Cr, and Zn) using atomic absorption spectrophotometer (Flame & graphite furnace system-PG990, UK).

N. Volatile Organic Compound (Gas chromatography–mass spectrometry).

Gas Chromatography–Mass Spectrometry (GC–MS) analysis was performed by using a GC (7890A, Agilent Technologies, Santa Clara, CA, USA) coupled with an MS (5975C Network, Agilent Technologies). The GC had an HP5MS column (non-polar column, Agilent Technologies), 30 m × 0.25 mm internal diameter and 0.25 μm film thickness. The carrier gas was helium flowing at a rate of 1.2 mL/minute. The initial oven temperature was 40 °C held for 3 min. It was raised to 200 °C at 3 °C/minute and then 16 °C/minute until it reached 240 °C and held at this temperature for 1.2 min. Mass spectra were recorded in a scanning range of 24–360 m/z. Enhanced ChemStation (ver. E.02.02.1431, Agilent Technologies) was used for identification by comparing the mass spectra of VOCs with those in the database of NIST11 (Gaithersburg, MD, USA) and Wiley 7N (John Wiley, NY, USA). The linear retention indices (LRI) of the VOCs were calculated using the retention times of n-alkane series from C9 to C20 as reference compounds. The calculated LRI were then compared to those in NIST web book. Each peak area of characterized compound was compared to the peak area of the internal standard (5 μL of 1 mg/mL benzophenone), while the VOCs were expressed as absent or present.

## 4. Conclusions

It can be seen that the green honey produced on Banggi Island off the coast of Kudat, Sabah, has a unique nutritional profile and colour. It is pertinent to highlight here that reports on green honey are greatly lacking, and we know very little regarding the nutritional profile of this honey produced by sting bees residing in underground nests. Although the honey’s green colour might appear unusual to the general public, the unique nutritional profile uncovered by this study might contribute to the body of knowledge on the global honey profile and garner the interest of food enthusiasts. The study found that the honey’s green colour was due to an unusually high chlorophyll content, a feature unseen in other honey samples. The nutritional and physicochemical data authenticated the purity of this green honey produced by these underground dwelling bees, despite the unusually higher sucrose content. This outcome might be due to underdeveloped or immature honey harvesting. Most importantly, the green honey is devoid of harmful substances, such as heavy metals, suggesting that Banggi Island is pollution free and the honey is safe for consumption. Meanwhile, the honey’s appreciably high content of three essential amino acids, namely, L-leucine, L-tryptophan, and L-histidine, signifies the importance of the commodity as a good source of these essential amino acids, whereas the exceptionally high trans-4-hydroxyproline content in raw green honey points to its role as an excellent building block for collagen synthesis in the body. Future studies employing metagenomic analysis might shed light on the origin of the chlorophyll, which imparts the honey’s green colour, as well as identify the presence of macro and microorganisms.

## Figures and Tables

**Table 1:** Physicochemical analyses of green honey compared to common types of raw honey from various origins.

Honey Types	Moisture (g/100 g)	pH	Free Acidity (meq/kg)	Ash (g/100 g)	Electric Conductivity (mS/cm)	HMF (mg/kg)	Total Phenolic Content (mg GAE/100 g)	Total Flavonoid Content (mg QE/100 g)	Antioxidant (DPPH) (%)	Chlorophyll (mg/kg)
Raw green	22.5 ± 0.55	3.4 ± 0.03	33.0 ± 6.145	0.08 ± 0.01	1.93 ± 0.02	0.01 ± 0.14	18.95 ± 0.08	7.6 ± 0.01	2.3 ± 0.02	539 ± 1.04
Processed green	12.7 ± 0.22	3.7 ± 0.02	28.0 ± 5.14	4.4 ± 0.01	0.85 ± 0.05	0.6 ± 0.25	15.57 ± 0.11	4.58 ± 0.01	2.0 ± 0.02	41 ± 0.44
Raw tualang	18.3 ± 0.21	3.5 ± 0.01	29.5 ± 6.14	1.3 ± 0.02	5.06 ± 0.02	6.4 ± 0.11	17.86 ± 0.02	6.92 ± 0.02	2.4 ± 0.03	ND
Tualang [4]	23.56 ± 0.32	3.2 ± 0.04	76.2 ± 4.14	0.15 ± 0.01	0.83 ± 0.01	46.54 ± 0.01	31.84 ± 0.76	40.5 ± 1.6	30.83 ± 0.01	ND
Gelam [4]	25.79 ± 0.21	3.3 ± 0.02	58.5 ± 4.14	0.28 ± 0.01	0.43 ± 0.01	45.42 ± 0.02	17.73 ± 0.86	58.3 ± 1.6	76.29 ± 0.02	ND
Kelulut [4]	28.3 ± 0.12	3.0 ± 0.03	448.10 ± 4.14	0.25 ± 0.01	1.05 ± 0.01	45.93 ± 4.72	42.6 ± 1.4	52.3 ± 3.4	71.93 ± 0.01	ND
Manuka [13]	19.5 ± 0.3	4.03 ± 0.2	50.0 ± 5.14	0.30 ± 0.01	0.53 ± 0.01	63.42 ± 0.01	1288.0 ± 102.8	37.64 ± 7.2	18.69 ± 0.9	ND

MF: hydroxymethylfurfural; DPPH: 2, 2-diphenyl-1-picryl-hydrazyl-hydrate; ND: not detected; GAE: gallic acid equivalents; QE: quercetin equivalents. Data presented as means ± standard deviation of three replicates. Raw Tualang is collected from Sabah (Tualang Sabah).

**Table 2:** Sugar analysis of green honey compared to common honey from various origins.

Honey Types	Total Sugar (g/100 g)	Sucrose (g/100 g)	Glucose (g/100 g)	Fructose (g/100 g)	Maltose (g/100 g)
Raw green	39.30 ± 0.02	14.55 ± 0.01	23.70 ± 0.05	11.35 ± 0.02	<10
Processed green	52.84 ± 0.07	27.72 ± 0.02	17.52 ± 0.03	19.56 ± 0.03	<10
Raw tualang	38.60 ± 0.02	ND	20.80 ± 0.01	26.9 ± 0.01	ND
Tualang [55]	63.40 ± 0.01	1.48 ± 0.01	28.79 ± 0.08	31.73 ± 0.14	0
Gelam [55]	62.74 ± 0.06	1.13 ± 0.03	29.17 ± 0.03	30.96 ± 0.03	1.48 ± 0.01
Kelulut [55]	62.70 ± 0.12	0.93 ± 0.04	29.72 ± 0.06	30.53 ± 0.01	1.52 ± 0.06
Manuka [55]	64.19 ± 2.69	1.32 ± 0.01	27.81 ± 1.20	34.14 ± 1.47	0.90 ± 0.05

Note: All values are expressed as mean ± standard deviation of three replications.

**Table 4:** Analysis of metal content in green honey compared with honeys from various origins.

Type of Honey	Arsenic (mg/kg)	Lead (mg/kg)	Nickel (mg/kg)	Cadmium (mg/kg)	Copper (mg/kg)	Cobalt (mg/kg)
Raw green	<0.5	<0.5	<0.5	ND	ND	ND
Processed green	<0.5	<0.5	<0.5	ND	ND	ND
Tualang [14]	0.062 ± 0.007	0.183 ± 0.007	ND	ND	1.25 ± 0.63	0.033 ± 0.002
Gelam [14]	0.064 ± 0.005	0.777 ± 0.012	ND	0.05 ± 0.07	2.21 ± 0.02	0.082 ± 0.005
Kelulut [14]	0.027 ± 0.001	0.691 ± 0.002	ND	0.78 ± 0.04	ND	ND
Manuka [14]	ND	ND	ND	ND	ND	ND

Note: All values are expressed as mean ± standard deviation of three replications.

**Table 5:** Volatile composition in green honey compared with Tualang.

Volatile Organic Compound	Processed Green Honey	Tualang Raw Honey	Tualang [71]
Ethyl Acetate (CH_3_COOC_2_H_5_)	Present	Present	Absent
Propanamide, N,N-dimethyl (C_5_H_11_NO)	Present	Absent	Absent
D-Limonene (C_10_H_16_)	Present	Absent	Absent
1,4-Cyclohexadiene, 1-methyl-4-(1-methylethyl) (C_10_H_16_)	Present	Absent	Absent
Cyclohexene, 1-methyl-4-(1-methylethylidene) (C_10_H_16_)	Present	Absent	Absent
Benzene, (2-methyl-1-propenyl)-o-Isopropenyltoluene (C_10_H_12_)	Present	Absent	Absent
Cyclotetradecane (C_14_H_28_)	Absent	Absent	Present
Hexadecanoctadecane (C_16_H_34_)	Absent	Absent	Present
Cycloeicosane (C_20_H_40_)	Absent	Absent	Present

## Data Availability

The data presented in this study are available upon request from the corresponding author.
